# Interpersonal interactions and empathy modulate perception of threat and defensive responses

**DOI:** 10.1038/srep19353

**Published:** 2016-02-03

**Authors:** C. Fossataro, C. F. Sambo, F. Garbarini, G. D. Iannetti

**Affiliations:** 1 Department of Neuroscience, Physiology and Pharmacology, University College London (UCL),United Kingdom; 2 SAMBA (SpAtial, Motor & Bodily Awareness) Research Group, Department of Psychology, University of Turin, Italy

## Abstract

The defensive peripersonal space (DPPS) is a vital “safety margin” surrounding the body. When a threatening stimulus is delivered inside the DPPS, subcortical defensive responses like the hand-blink reflex (HBR) are adjusted depending on the perceived threat content. In three experiments, we explored whether and how defensive responses are affected by the interpersonal interaction within the DPPS of the face. In Experiment 1, we found that the HBR is enhanced when the threat is brought close to the face not only by one’s own stimulated hand, but also by another person’s hand, although to a significantly lesser extent. In Experiments 2 and 3, we found that the HBR is also enhanced when the hand of the participant enters the DPPS of another individual, either in egocentric or in allocentric perspective. This enhancement is larger in participants with strong empathic tendency when the other individual is in a third person perspective. These results indicate that interpersonal interactions shape perception of threat and defensive responses. These effects are particularly evident in individuals with greater tendency to having empathic concern to other people.

The defensive peripersonal space (DPPS) is a vital “safety margin” surrounding the body. The DPPS has a crucial role for survival: whenever a salient and potentially dangerous stimulus approaches or enters it, the individual engages in more efficient actions aimed at self-protection^1^,^2^. We have recently identified a DPPS in humans by recording a defensive reflex response – the eye blink elicited by hand stimulation (hand-blink reflex, HBR).

The HBR as recorded in this study may not reflect a typical startle response. Indeed, although the blink reflex is a consistent component of the startle reaction, this does not imply that it is necessarily part of a startle response. Given that startle responses immediately and dramatically habituate in response to rhythmic stimulation, the lack of such habituation of the HBR in response to repeated stimulation (i.e. 1 stimulus every 30 seconds, see Fig. 1 in Sambo *et al*. 2012a^3^) indicate that the HBR cannot be entirely considered of startle origin.

The HBR is dramatically increased when the hand is located close to the face^3^,^4^. We have suggested that in this condition the electrical stimulation of the hand is coded as a sensory event potentially dangerous for the eye, thus resulting in a larger HBR^3^,^5^.

The HBR enhancement may result from the modulation of the brainstem circuits subserving the HBR by associative cortical areas (such as the premotor cortex and the ventral intraparietal area) involved in representing the peripersonal space and in detecting potentially dangerous stimuli near the body^6^,^7^.

Importantly, the excitability of the HBR brainstem circuits is finely adjusted in a purposeful manner depending on the context in which the threat is applied. For example, the strength of the HBR enhancement is linearly related to the probability of occurrence of potentially dangerous stimuli close to the face^5^. Furthermore, the presence of a defensive object in front of the face can dramatically reduce the DPPS size, to an extent that the HBR enhancement observed when the hand is close to the face is abolished when a thin screen is interposed between the hand and the eye^3^.

The multisensory representation of the peripersonal space allows close interactions not only with objects, but also with other individuals^8^,^9^,^10^,^11^. How do interpersonal interactions affect the perception of threat within one’s own or someone else’s DPPS? This question is important as interactions between individuals are a hallmark of primate social life. Through social interactions the DPPS of different individuals overlap in space. This idea raises a series of interesting questions, related to how defensive responses are affected by the presence of threats brought close to one’s body by other individuals.

Here we addressed these questions in three experiments. In Experiment 1, we tested whether the proximity of the threat to the face may enhance the magnitude of the HBR irrespective of whether such threat is brought by one’s own arm or the arm of another person. In Experiment 2, we tested whether the magnitude of the HBR is also enhanced when the participant’s own hand enters the DPPS of another individual. In Experiment 3, we tested whether a possible HBR modulation observed when the participant’s own hand is close to the face of another individual may depend on that, in that posture, the hand is also inside the participant’s own DPPS. We did this by manipulating the perspective (either egocentric or allocentric) of the other’s face toward witch the threat was directed.

## Results

### Experiment 1

**Figure 1:**
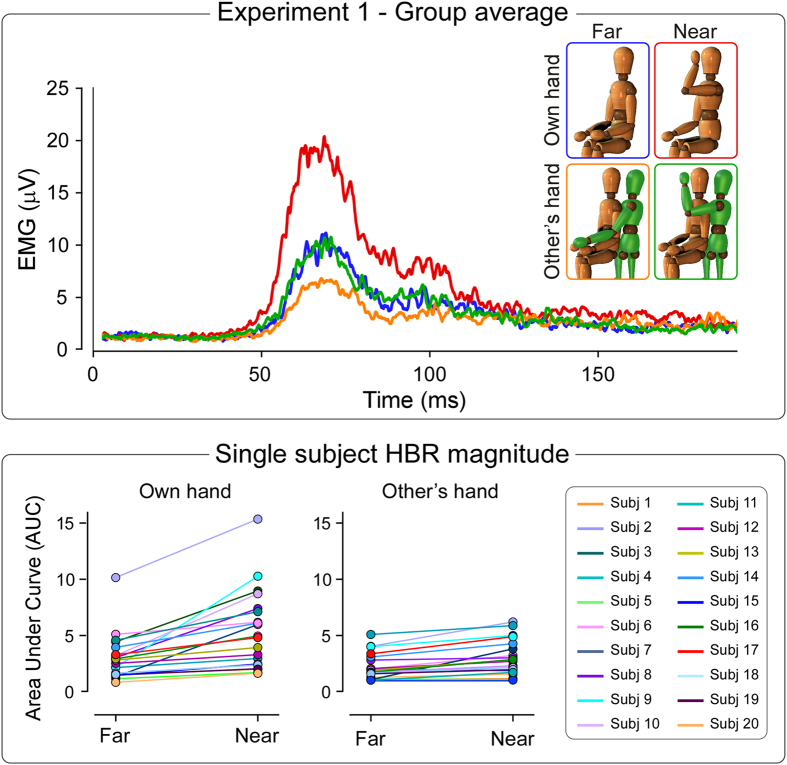
Experiment 1. *Top*. Group-average, rectified HBR waveforms. In the condition ‘Own hand’, the HBR was elicited by stimulating the participant’s hand in the ‘far’ (blue) and ‘near’ (red) position. In the condition ‘Other’s hand’, the HBR was elicited by stimulating the participant’s hand always in the ‘far’ position, while the other person’s hand was located either ‘far’ (orange) or ‘near’ (green) from the participant’s face. *Bottom*. Single-subject HBR magnitudes (AUC, arbitrary units) in the far and near positions, for the own hand and the other person’s hand conditions. There was a significant interaction between the factors ‘hand position’ and ‘hand ownership’ (F_1,19_ = 12.6, p = 0.002), indicating that the HBR enhancement was significantly stronger when the threat was brought inside the DPPS by one’s own arm, compared to when it was brought by the arm of another person.

Figure 1 shows the HBR recorded in Experiment 1. In the condition ‘own hand’, the HBR was elicited by stimulating the participant’s hand in the ‘far’ and ‘near’ positions. In the condition ‘other’s hand’, the HBR was elicited by stimulating the participant’s hand always in the ‘far’ position, while the other person’s hand was located either outside (‘other’s hand far’) or inside (‘other’s hand near’) the participant’s DPPS of the face.

We observed main effects of both ‘hand position’ (F_1,19_ = 32.0, p = 0.00002) and ‘hand ownership’ (F_1,19_ = 13.9, p = 0.002). Crucially, we found a strong interaction between these two factors (F_1,19_ = 12.6, p = 0.002). This indicates that the HBR enhancement was significantly stronger when one’s own stimulated hand was inside the DPPS of the face (far: 3.0 ± 2.1, near: 5.4 ± 3.5; +93 ± 99%, p = 0.0001), compared to when the other person’s hand was inside the DPPS of the face (far: 2.2 ± 1.2, near: 3 ± 1.6; +41 ± 55%, p = 0.02) (Fig. 1).

### Experiment 2

**Figure 2:**
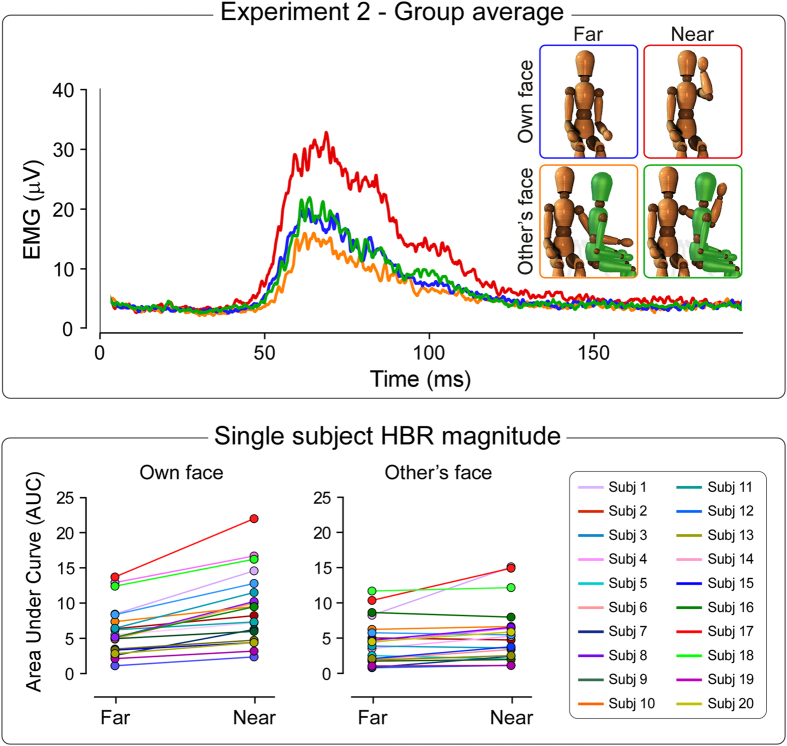
Experiment 2. *Top*. Group-average, rectified HBR waveforms. In the condition ‘own face’, the HBR was elicited by stimulating the participant’s hand in the ‘far’ (blue) and ‘near’ (red) position. In the condition ‘other’s face’, the HBR was elicited by stimulating the participant’s hand located either ‘far’ (orange) or ‘near’ (green) the other person’s face. *Bottom*. Single-subject HBR magnitudes (AUC, arbitrary units) in the far and near positions, for the own face and the other person’s face conditions. There was a significant interaction between the factors ‘hand position’ and ‘face ownership’ (F_1,19_ = 24.6, p < 0.0001), indicating that the HBR enhancement was significantly stronger when the threat was inside one’s own peripersonal space, compared to when it was inside the peripersonal space of the other person.

Figure 2 shows the HBR recorded in Experiment 2. In the condition ‘own face’ the HBR was elicited by stimulating the participant’s hand located either outside (‘far’) or inside (‘near’) the participant’s DPPS (as in the condition ‘own hand’ of Experiment 1). In the condition ‘other’s face’ the HBR was elicited by stimulating the participant’s hand located either outside (‘far’) or inside (‘near’) the other person’s DPPS of the face.

We observed a strong main effect of ‘hand position’ (F_1,19_ = 32.0, p < 0.0001) and ‘face ownership’ (F_1,19_ = 15.3, p = 0.001). Crucially, we found a strong interaction between these two factors (F_1,19_ = 24.6, p < 0.0001). This indicates that the HBR enhancement was significantly stronger when the stimulated hand was inside one’s own peripersonal space of the face (far: 5.7 ± 3.5, near: 8.9 ± 5.1; +70 ± 51%, p = 0.0001), compared to when it was inside the peripersonal space of the other’s face (far: 4.5 ± 3.1, near: 5.7 ± 4.1; +35 ± 48%, p = 0.001) (Fig. 2).

### Experiment 3

**Figure 3:**
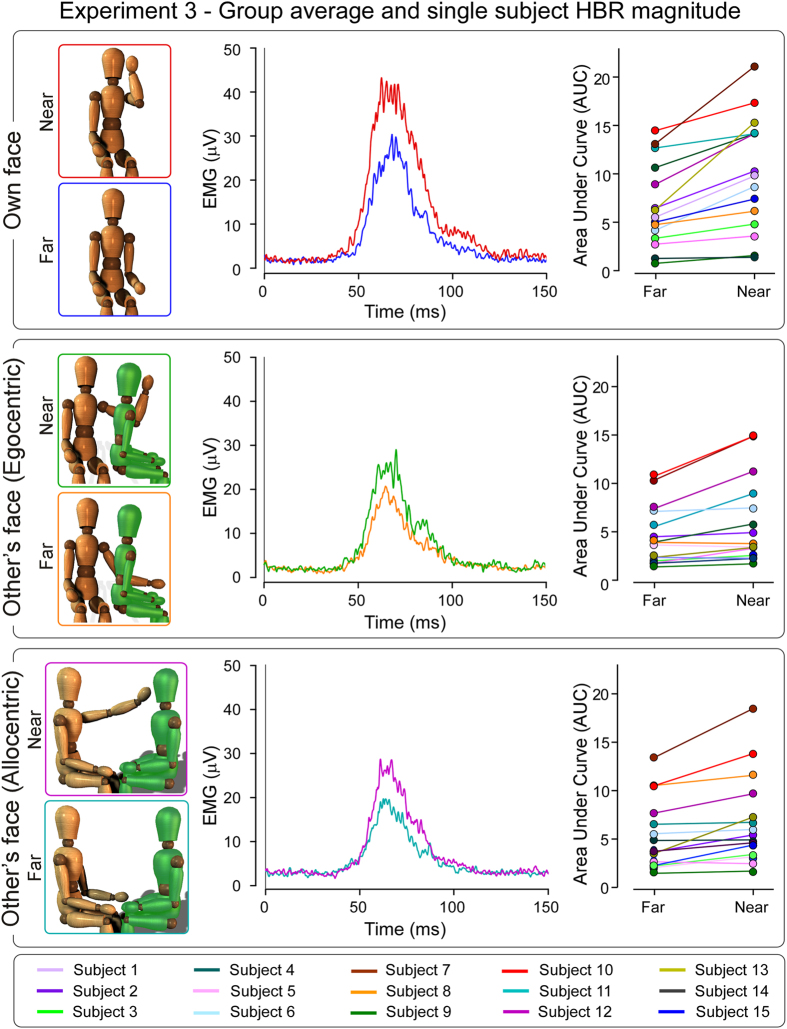
Experiment 3. Group-average, rectified HBR waveforms. In the condition ‘own face’ (top panel), the HBR was elicited by stimulating the participant’s hand in the ‘far’ (blue) and ‘near’ (red) position. In the condition ‘other’s face egocentric’ (middle panel), the HBR was elicited by stimulating the participant’s hand located either ‘far’ (orange) or ‘near’ (green) the other person’s face, in a first person perspective. In the condition ‘other’s face allocentric’ (bottom panel), the HBR was elicited by stimulating the participant’s hand located either ‘far’ (cyan) or ‘near’ (purple) the other person’s face, in a third person perspective. Single-subject HBR magnitudes (AUC, arbitrary units) in the far and near positions are shown for each condition. There was a significant interaction between the factors ‘hand position’ and ‘condition’ (F_2,28_ = 8.0, p = 0.002), indicating that the HBR enhancement was stronger when the threat was inside one’s own peripersonal space, compared to when it was inside the peripersonal space of the other person, either in egocentric or in allocentric perspective.

Figure 3 shows the HBR recorded in Experiment 3. In the condition ‘own face’, the HBR was elicited by stimulating the participant’s hand located either outside (‘far’) or inside (‘near’) the participant’s DPPS (as in the condition ‘own hand’ of Experiments 1 and 2). In the condition ‘other’s face egocentric’, the HBR was elicited by stimulating the participant’s hand located either outside (‘far’) or inside (‘near’) the other person’s DPPS of the face from a first person (i.e. egocentric) perspective. In the condition ‘other’s face allocentric’ the HBR was elicited by stimulating the participant’s hand located either outside (‘far’) or inside (‘near’) the other person’s DPPS of the face from a third person (i.e. allocentric) perspective.

*Effect of face ownership and hand position on the HBR magnitude*. We observed main effects of both ‘hand position’ (F_1,14_ = 25.3, p = 0.0002) and ‘condition’ (F_2,28_ = 8.4, p = 0.0001). Crucially, we found a strong interaction between these two factors (F_2,28_ = 8.0, p = 0.002). Despite the fact that HBR ‘far’-‘near’ enhancement was significant in all three conditions, the interaction indicates that the enhancement was significantly stronger when the stimulated hand was inside the one’s own DPPS of the face (far own: 6.6 ± 4.3, near own: 10.0 ± 6; +55 ± 38%, p = 0.00005), compared to when was placed inside the other person’s DPPS of the face either in a first person perspective (far other’s face egocentric: 4.6 ± 3.0, near other’s face egocentric: 6.1 ± 4.4; +31 ± 27%, p = 0.001), or in a third person perspective (far other’s face allocentric: 5.2 ± 3.6, near other’s face allocentric: 6.8 ± 4.6; +34 ± 32%, p = 0.0008). Crucially, when the hand was placed near to other’s face in the first or in the third person perspective the magnitude of the HBR was not significantly different (other’s face egocentric: 6.1 ± 4.4; other’s face allocentric: 6.8 ± 4.6; p = 0.2) (Fig. 3).

*Correlation between HBR enhancement and empathic traits*. To explore the correlation between HBR enhancement and empathic traits, after the HBR recording participants completed the Interpersonal Reactivity Index (IRI)^12^. The IRI is a self-report multidimensional psychometric measure composed of 28 items designed to measure both cognitive and emotional components of empathy. Participants rated each item on a 5-point Likert scale ranging from “Does not describe me well” to “Describe me very well”. The measure has 4 subscales, each made up of 7 different items. These subscales are: Perspective Taking (e.g. “When I am upset at someone, I usually try to ‘put myself in his shoes’ for a while”); Fantasy Scale (e.g. “I really get involved with the feelings of the characters in a novel”); Empathic Concern (e.g. “When I see someone being taken advantage of, I feel kind of protective towards them”); Personal Distress (e.g. “In emergency situations, I feel apprehensive and ill-at-ease”). Each subscale score ranges from 0 to 28.

**Figure 4:**
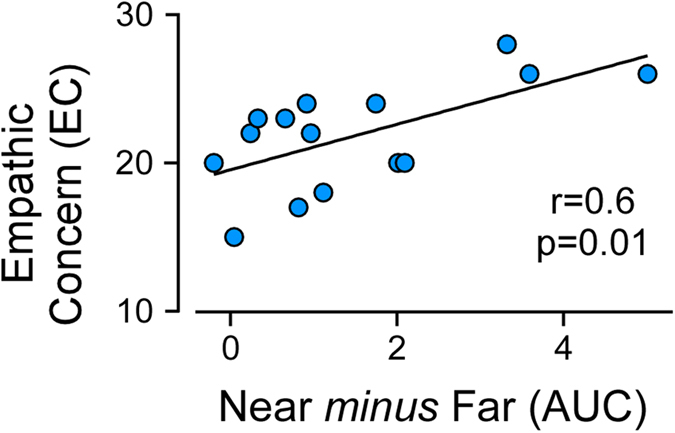
Correlation between HBR enhancement and empathic traits. There was a significant positive relationship between the ‘far-near’ increase observed when the hand was inside the other person’s peripersonal space in a third person perspective and the self-report measures of Empathic Concern (EC; r = 0.6, p = 0.012).

When the stimulated hand entered the other person’s DPPS in a third person perspective, we observed a significant positive relationship between the ‘far-near’ increase and self-report measures of Empathic Concern (r = 0.6, p = 0.01), as well as a trend towards a positive relationship between the ‘far-near’ increase and self-report measures of Perspective Tacking: r = 0.5, p = 0.05. The other two correlations between the ‘far’-‘near’ increase and each of the other two IRI subscales were not significant (Fantasy Scale: r = 0.4, p = 0.2; Personal Distress: r = 0.4, p = 0.1) (Fig. 4). On the contrary, there was no evidence of a relationship between the ‘far-near’ increase and self-report measures of empathic trait when the stimulated hand entered the other’s DPPS in first person perspective (Empathic Concern: r = −0.3, p = 0.2, Fantasy Scale: r = 0.2, p = 0.5; Perspective Tacking: r = 0.3, p = 0.2; Personal Distress: r = −0.3, p = 0.3).

## Discussion

In this study we tested whether the physical proximity with other individuals affects one’s own defensive responses when threatening stimuli are delivered inside the DPPS of the face. We observed three main findings. First, the HBR is enhanced not only when one’s own stimulated hand enters the DPPS of the face, but also when another person’s hand is placed inside the participant’s DPPS of the face, although to a significantly lesser extent (Experiment 1). Second, participants show an enhanced HBR when their stimulated hand enters the DPPS of the face of another individual (Experiments 2 and 3). Third, participants with stronger empathic trait tended to have a larger HBR enhancement when their stimulated hand enters the DPPS of the face of another individual positioned in a third person perspective. These results indicate that high-level cognitive processes related to assessing the threat of confronting another individual result in a fine modulation of subcortical circuits mediating defensive reflex responses.

In Experiment 1 we tested whether the proximity of the threat to the face may enhance the magnitude of the HBR irrespective of whether such threat is brought by one’s own hand or the hand of another person.

We confirmed that the HBR is significantly enhanced, by approximately a factor of two, when one’s own stimulated hand enters the DPPS of the face^3^,^5^. An important new finding of the present study is that the HBR is enhanced even when another person’s hand is placed inside one’s own DPPS, while one’s own hand is placed outside the DPPS. This finding indicates that the top-down facilitation of a protective reflex occurs more when the threat, approaching the body territory to be defended, is brought not only by one’s own hand, but also by another person’s hand.

However, when another person’s hand is placed inside one’s own DPPS, the HBR enhancement is significantly smaller (Fig. 1). This is likely to be explained by the conflicting threat information present in this condition. Indeed, while the proprioceptive system carries ‘safety information’ signalling that the threat (i.e. the electrical stimulus triggering the HBR) is not close to the eye, the visual system carries ‘threat information’, signalling that the threat (i.e. the electrical stimulus delivered to the other person’s hand) is close to the eye. Therefore, the observation that the HBR enhancement is significantly smaller when another person’s hand is placed inside one’s own DPPS indicates that, when there is a mismatch between ‘safety’ and ‘threat’ information, the ‘threat’ information carried by the visual system is modulated by the ‘safety’ information carried by the somatosensory system. This results in a reduced enhancement of the HBR magnitude (Fig. 1). An additional explanation for the smaller HBR increase observed when another person’s hand was placed inside one’s own DPPS relates to the social context of the experiment. This may have primed the participants to passively inhibit their concern about the potential danger represented by the other person’s hand, or to presume that the experimenters would ensure safety. In any case, such possible context-dependent inhibition of the concern about the potential danger of the other person’s hand is compatible with the interpretation of a sensory safety-danger mismatch.

In previous studies we provided evidence that the HBR enhancement is mediated by a tonic, top-down modulation of the excitability of brainstem circuits specifically receiving input from the stimulated hand^3^. We also showed that the strength of this modulation is dependent on the probability of occurrence of a threatening stimulus close to the body district to protect^5^; in other words, the amount of top-down facilitation is dependent on the assessment of the threat situation. Such threat level-dependent facilitation makes functional sense, since the HBR increase has a clear metabolic and behavioural cost, which the system is ready to meet only when there is a reasonable certainty that such cost has a value in terms of avoiding damage to the body^5^. In the context of the current experiment, when another person’s hand is placed close to the participant’s eye while the participant’s hand is located outside their own DPPS (Fig. 1), the mismatch between ‘safety’ and ‘threat’ information may explain why the HBR enhancement is smaller in this condition compared to when the participant’s own hand is placed inside the DPPS. However, and importantly, the observation that the HBR is enhanced even when another person’s hand is inside the participant’s own DPPS indicates that there is a contribution of both the proprioceptive and the visual system in determining the perceived level of threat (and the functional advantage of meeting the cost of establishing the top down facilitation). Thus, the threat assessment resulting in the HBR enhancement is sensitive to sensory information carried by other modalities, besides proprioception.

In Experiment 2 we tested whether the magnitude of the HBR is also enhanced when the participant’s hand enters the DPPS of another individual. We found that the HBR is indeed enhanced when the participant’s stimulated hand enters the DPPS of the face of another individual, although such enhancement is significantly less strong compared to when the hand enters one’s own DPPS (Fig. 2).

Importantly, in Experiment 2, both the participant and the other person had the same perspective. This implies that the participant’s DPPS of the face might have partly overlapped with the other person’s face DPPS. Therefore, we could not rule out that the observed HBR increase when the participant’s hand was close to the face of the other person could be due to the fact that, in that posture, the hand was also inside the participant’s own DPPS.

We addressed this issue in Experiment 3, by testing whether the HBR magnitude is enhanced irrespective of whether the participant’s hand enters the DPPS of another individual in egocentric perspective (i.e. a condition in which the two DPPS could be partially overlapping) or in allocentric perspective (i.e. a condition in which the two DPPS are not overlapping) (Fig. 3, left panels). The results of Experiment 3 confirmed that the HBR is enhanced also when the participant’s hand enters the DPPS of another individual in allocentric perspective. This observation indicates that the HBR enhancement observed both in the ‘other’s face’ condition of Experiment 2 and in the ‘egocentric’ condition of Experiment 3 is determined by the participant’s assessment of the other person’s risk. Therefore, it would make sense that in individuals with a high tendency to having concern for the others (i.e. participants with a high empathic trait), this information results in a stronger enhancement of their own HBR. Indeed, when the stimulated hand entered the other person’s DPPS in a third person perspective (i.e. in a face-to-face perspective, Fig. 3), the ‘far-near’ increase was larger in individuals with high self-reported measures of Empathic Concern (Fig. 4). It is worth noting that the lack of a difference in HBR between placing one’s own hand in another person’s peripersonal space when facing them (a more classically confrontational orientation compared with the egocentric posture) underlines the specificity of the HBR response to defense. Likewise, the stronger link between the far-near increase of HBR and the Empathic Concern scale relative to the Personal Distress scale may also indirectly suggest that the HBR increases observed in Experiments 2 and 3 are not related to generic arousal at the prospect of touching another person, but to the empathic defensiveness of the other person.

It has been suggested that the observation of someone else in pain can produce painful sensations in the onlooker^13^, and that such observation activates multimodal brain areas that are also activated during physical painful stimulation^14^,^15^. Activity in these areas is known to be largely unspecific for nociception and pain, and, instead, probably related to attentional orienting toward salient sensory stimuli, regardless of their sensory modality^16^,^17^. Importantly, a subset of these areas contain representations not only of one’s own peripersonal space, but also of the peripersonal space of other individuals (for a review see Brozzoli *et al*.^18^). In turn, the presence and interaction with other individuals affect the cortical representations of PPS, and shape its boundaries^19^. Furthermore, parietal visuo-tactile neurons in non-human primates fire when visual stimuli approach both the experimenter’s and the monkey’s body, giving further support to the idea that the perception of the peripersonal space of other individuals exploits the same representations of one’s own peripersonal space^10^,^20^.

Our current observation that individuals with a strong empathic trait have an enhanced HBR when their stimulated hand enters the DPPS of the face of another individual (Fig. 3), indicates that the tonic, top-down facilitation of the brainstem HBR circuits can be also activated by the awareness of the proximity between a physical threat and someone else’s face. What could be the functional significance of the increase of one’s own defensive responses when observing someone else in physical danger? Evolutionary theories suggest that empathy, that is, the propensity for feeling the emotions of others and for having concern for their wellbeing, is an ancient phylogenetic mechanism, deeply-rooted in human nature^21^. The ability to empathize, especially in the context of threat and pain, allows learning about potentially dangerous environmental events, and motivates altruism and prosocial behaviors^22^. The observed increase in one’s own HBR when their hand enters the other person’s DPPS, may be consequent to the *embodiment of others’ perception of threat*, that is, the activation of one’s sensorimotor representation caused by observing others in danger. This mirror response may facilitate the motor implementation of the observer’s defensive responses before stimuli signalling a threat for one’s own body are actually detected by the somatosensory system^23^.

Although the laboratory-specific experimental setting (and, in particular, the electrical nature of the stimulus) might reduce the generalization of this result to more ecological environments, our observation further suggests that the tendency to embrace another person’s psychological perspective also extends to adopting another person’s physical point of view. Indeed, given that the increase in HBR magnitude exquisitely relies on the spatial location of the threat in egocentric coordinates^24^, the observation of an HBR enhancement in the onlookers (particularly in those with a high Empathic Concern score) implies that they remap their own system of egocentric coordinates and align it with that of the other person.

## Methods

### Participants

Twenty healthy volunteers (20–39 years, mean ± SD 25.2 ±3.6; 10 females) participated in Experiment 1. A separate group of twenty volunteers (20–28 years, 23.7 ± 2.5; 11 females) participated in Experiment 2. Another group of fifteen volunteers (21–27 years, 23.3 ± 2.1; 9 females) participated in Experiment 3. All participants were right-handed, naïve to the experimental procedure, and gave written informed consent before taking part in the study, which was approved by the University College London’s ethics committee. The experimental procedures were carried out in accordance with the approved guidelines.

All three Experiments involved the use of one “other person” besides the volunteer. The “other person” was not always the same individual. Typically, the person in the role of “the other” was another volunteer, who did not participate in the experiment as main subject. However, when we were not able to find a volunteer available, a research assistant or another lab member (i.e. a confederate of the researcher) assumed the role of “the other”. The “other person” was always presented to the participant as a fellow participant to the experiment. Given the relevance of sex to both perceived threat of violence and to the desirability of physical contact, we kept the sex and the race of the subject and of the “other person” the same. Race differences are specified above. In Experiments 1 and 2, the sex of the pair was always the same. In Experiment 2 there was only one pair in which the subject was a male and the “other person” a female. In Experiment 1 all pair were of the same race (white Caucasian). In both Experiments 2 and 3 there was one pair including one non-Caucasian volunteer.

### Stimulation and recording

Transcutaneous electrical stimuli consisted in constant current square-wave pulses (DS7A, Digitimer) delivered to the left median nerve at the wrist, using a surface bipolar electrode. Stimulus duration was 200 μs and the inter-stimulus interval was ~30 s. Stimulus intensity was adjusted, in each participant, to elicit clear and reproducible HBR responses (as in^3^,^4^,^5^). Electromyographic (EMG) activity was recorded from the *orbicularis oculi* muscles, using two pairs of bipolar surface electrodes, with the active electrode over the mid lower eyelid and the reference electrode laterally to the outer canthus. Signals were amplified and digitized at 8,196 Hz (ISA 1004, Micromed).

### Procedures

Participants were seated comfortably. The stimulus intensity was adjusted, in each participant, to elicit a clear HBR in three consecutive trials. Mean stimulus intensities were 43 ± 17 mA, range 20–99 mA (Experiment 1), 34 ± 15 mA, range 20–60 mA (Experiment 2), and 32 ± 18 mA, range 20–80 mA (Experiment 3).

#### Experiment 1

To investigate whether the HBR elicited by the stimulation of the participant’s hand is modulated by the presence of another person’s hand inside the participant’s DPPS of the face, we recorded the HBR in two conditions. In the condition ‘own hand’, the HBR was elicited by stimulating the participant’s hand in the ‘far’ and ‘near’ positions, in alternating trials (as in Sambo *et al*., 2012a^3^). In the ‘far’ position participants were sitting with their forearm resting on a pillow and the left hand close to the ipsilateral knee, at a distance of ~60 cm from the ipsilateral side of their face. In the near position, participants were sitting with their left arm resting on a chair arm, holding the wrist at ~4 cm from the ipsilateral side of their face, namely inside the DPPS of the face. In the condition ‘other’s hand’, the HBR was elicited by stimulating the participant’s hand always in the ‘far’ position, while the other person’s hand was located either outside (‘far’) or inside (‘near’) the participant’s DPPS, in alternating trials. A transcutaneous electrical stimulator, identical to the one used to stimulate the participant’s hand, was attached to the other person’s wrist. In both position, the participant’s hand was always kept in the far position, and covered with a light towel. The presence of the towel is a parsimonious explanation for the smaller HBR magnitude in the ‘other’s hand’ condition, given that the presence of objects between the stimulated hand and the eye reduces the HBR magnitude^5^. The position of the other person’s arm was congruent with respect to the participant’s trunk midline. In the ‘far’ position, the other person’s hand was resting on a pillow close to the participant’s ipsilateral knee, so that the other person’s hand was clearly outside the DPPS of the participant’s face; in the ‘near’ position the other person’s hand was inside the DPPS of the participant’s face, at a distance of ~4 cm from the participant’s face. Participants were clearly informed that the other person’s hand was electrically stimulated. The participant’s right hand was never stimulated and the arm was held along the body during the experiment. The experiment consisted of four recording blocks: two blocks for the ‘own hand’ condition, and two blocks for the ‘other’s hand’ condition. The order of blocks was balanced across participants: half of the participants started the experiment with the ‘own hand’ condition, and the other half with the ‘other’s hand’ condition. In each block 16 stimuli were delivered: 8 to the hand in the far position, and 8 to the hand in the near position, in alternating trials.

#### Experiment 2

To investigate whether the HBR is modulated by the position of the participant’s hand in respect to another person’s face, we recorded the HBR in two conditions. In the condition ‘own face’, the HBR was elicited by stimulating the participant’s hand located either outside (‘far’) or inside (‘near’) the participant’s DPPS (as in the condition ‘own hand’ of Experiment 1). In the condition ‘other’s face’, the HBR was elicited by stimulating the participant’s hand located either outside (‘far’) or inside (‘near’) the other person’s DPPS of the face. The participant’s left hand was aligned with the other person’s left shoulder, in a congruent position with respect to the other person’s trunk midline. In both positions, the other person’s left hand, which was always stimulated, rested on his/her own thigh, close to the ipsilateral knee and hidden by a towel. Participants were informed that the other person’s hand was stimulated. In the far position, the participant’s hand was resting on a pillow close to the other person’s ipsilateral knee (i.e. clearly outside the DPPS of the other person’s face). In the near position the participant’s hand was inside the DPPS of the other person’s face. The right hand was never stimulated and the arm was resting along the body during the experiment. The experiment consisted of four recording blocks: two blocks for the ‘own face’ condition and two blocks for the ‘other’s face’ condition. The order of blocks was balanced across participants: half of the participants started the experiment with the ‘own face’ condition, and the other half with the ‘other’s face’ condition. In each block 16 stimuli were delivered: 8 to the hand in the far position, and 8 to the hand in the near position, in alternating trials.

#### Experiment 3

To investigate whether the HBR magnitude is enhanced irrespectively of whether the stimulated hand entered the other person’s DPPS either in a first person (i.e. egocentric) perspective or in a third person (i.e. allocentric) perspective, we recorded the HBR in three conditions. In the condition ‘own face’, the HBR was elicited by stimulating the participant’s hand located either outside (‘far’) or inside (‘near’) the one’s own DPPS (as in the condition ‘own hand’ of Experiment 1). In the condition ‘other’s face egocentric’, the HBR was elicited by stimulating the participant’s hand located either outside (‘far’) or inside (‘near’) the other person’s DPPS of the face, in a first person perspective (as in the condition ‘other’s face’ of Experiment 2). In the condition ‘other’s face allocentric’, the HBR was elicited by stimulating the participant’s hand located either outside (‘far’) or inside (‘near’) the other person’s DPPS, in a third person perspective (i.e. with the two participants facing each other). In this condition, the stimulated hand was either kept in the ‘far’ position, or straight ahead, close to the other person’s face, and clearly outside the participant’s own DPPS. The other person’s left hand was never stimulated and always rested on his/her thigh, close to the ipsilateral knee and hidden by a towel. Participants were informed that the other person’s hand was stimulated. The experiment consisted of three recording blocks: one for the ‘own face’ condition, one for the ‘other’s face egocentric’ condition, and one for the ‘other’s face allocentric’ condition. The order of blocks was randomized across participants. In each block 16 stimuli were delivered: 8 to the hand in the far position, and 8 to the hand in the near position, in alternating trials.

### Data Analysis and Statistics

EMG signals were analysed using Letswave (http://nocions.org/letswave)^25^. EMG signals from each participant were high-pass filtered (55 Hz), full-wave rectified and averaged across ipsilateral and contralateral recording sides. In each experiment HBR responses were averaged separately according to condition, resulting in four waveforms (Experiment 1 and 2) or six waveforms (Experiment 3) for each subject. In each average waveform HBR magnitude was measured as area under the curve (AUC, arbitrary units).

In Experiment 1, we performed a two-way, repeated-measures ANOVA on the HBR magnitude, with ‘hand ownership’ (two levels: own hand, other’s hand) and ‘hand position’ (two levels: far, near) as experimental within-subject factors. In Experiment 2, we performed a two-way, repeated-measures ANOVA on the HBR magnitude, with ‘face ownership’ (two levels: own face, other’s face) and ‘hand position’ (two levels: far, near) as experimental within-subject factors. In Experiment 3, we performed a two-way, repeated-measures ANOVA on the HBR magnitude, as measured by the AUC, with ‘condition’ (three levels: own face, other’s face egocentric, other’s face allocentric) and ‘hand position’ (two levels: far, near) as experimental within factors. Duncan tests were used to perform post-hoc pairwise comparison.

To investigate whether individual differences in empathic traits predicted the HBR increase produced by the proximity between one’s own hand and the other person’s face, we correlated each of the four IRI subscales with the near-far enhancement of the HBR response in the ‘other’s face’ conditions. The threshold of significance of these correlations was set at 0.0125 (0.05/4 = 0.0125) to account for the multiple statistical comparisons.

## Additional Information

**How to cite this article**: Fossataro, C. *et al*. Interpersonal interactions and empathy modulate perception of threat and defensive responses. *Sci. Rep*. **6**, 19353; doi: 10.1038/srep19353 (2016).
